# RIG-I Activation by a Designer Short RNA Ligand Protects Human Immune Cells against Dengue Virus Infection without Causing Cytotoxicity

**DOI:** 10.1128/JVI.00102-19

**Published:** 2019-06-28

**Authors:** Victor Ho, Hui Yee Yong, Marion Chevrier, Vipin Narang, Josephine Lum, Ying-Xiu Toh, Bernett Lee, Jinmiao Chen, Ern Yu Tan, Dahai Luo, Katja Fink

**Affiliations:** a Singapore Immunology Network, Agency for Science, Technology and Research, Singapore; b School of Biological Sciences, Nanyang Technological University, Singapore; c Lee Kong Chian School of Medicine, Nanyang Technological University, Singapore; d Department of General Surgery, Tan Tock Seng Hospital, Singapore; Hudson Institute of Medical Research

**Keywords:** dengue, RIG-I, antiviral, human skin cell, innate activation, primary human cells, short hairpin RNA

## Abstract

Virus-derived double-stranded RNA (dsRNA) molecules containing a triphosphate group at the 5′ end are natural ligands of retinoic acid-inducible gene I (RIG-I). The cellular pathways and proteins induced by RIG-I are an essential part of the innate immune response against viral infections. Starting from a previously published RNA scaffold (3p10L), we characterized an optimized small dsRNA hairpin (called 3p10LG9, 25 nucleotides [nt] in length) as a highly efficient RIG-I activator. Dengue virus (DENV) infection in cell lines and primary human skin cells could be prevented and restricted through 3p10LG9-mediated activation of RIG‐I. This antiviral effect was RIG-I and interferon signal dependent. The effect was temporary and was reversed above a saturating concentration of RIG-I ligand. This finding revealed an effective feedback loop that controls potentially damaging inflammatory effects of the RIG-I response, at least in immune cells. Our results show that the small RIG-I activator 3p10LG9 can confer short-term protection against DENV and can be further explored as an antiviral treatment in humans.

**IMPORTANCE** Short hairpin RNA ligands that activate RIG-I induce antiviral responses in infected cells and prevent or control viral infections. Here, we characterized a new short hairpin RNA molecule with high efficacy in antiviral gene activation and showed that this molecule is able to control dengue virus infection. We demonstrate how structural modifications of minimal RNA ligands can lead to increased potency and a wider window of RIG-I-activating concentrations before regulatory mechanisms kick in at high concentrations. We also show that minimal RNA ligands induce an effective antiviral response in human skin dendritic cells and macrophages, which are the target cells of initial infection after the mosquito releases virus into the skin. Using short hairpin RNA as RIG-I ligands could therefore be explored as antiviral therapy.

## Abstract (precis)

Short hairpin RNA ligands that activate RIG-I induce antiviral responses in infected cells and prevent or control viral infections. Here, we characterized a new short hairpin RNA molecule with high efficacy in antiviral gene activation and showed that this molecule is able to control dengue virus infection. We demonstrate how structural modifications of minimal RNA ligands can lead to increased potency and a wider window of RIG-I-activating concentrations before regulatory mechanisms kick in at high concentrations. We also show that minimal RNA ligands induce an effective antiviral response in human skin dendritic cells and macrophages, which are the target cells of initial infection after the mosquito releases virus into the skin. Using short hairpin RNA as RIG-I ligands could therefore be explored as antiviral therapy.

## INTRODUCTION

The RIG-I like receptors (RLRs), including RIG-I, MDA5, and LGP2, detect viral infections and initiate interferon-dependent and -independent antiviral immune responses (1, 2). RIG-I is activated by the binding of an RNA substrate containing 5′-triphosphorylated short double-stranded RNA (dsRNA), although the absolute requirement for recognition is the basic duplex RNA. In contrast to RIG-I, MDA5 is activated by long double-stranded RNA (1, 3).

RIG-I recognizes viral RNA due to the presence of the triphosphorylated 5′ end, distinguishing replicating viruses from endogenous RNA that is further processed with the addition of a 5′ cap (4). In single-stranded RNA (ssRNA) viruses, the partially complementary, panhandle-structure terminal sequences are recognized by RIG-I (5–8). In addition to 5′-triphosphorylated RNA, RIG-I also binds to 5′-diphosphorylated RNA and Cap 0 RNA (9).

Upon binding of RNA to RIG-I, the activated RIG-I binds to MAVS (alternative names are IPS-1, Cardiff, and VISA). This leads to the activation of transcription factors IRF3/IRF7 and NF-κB, which trigger the production of type I interferon (IFN) and other antiviral mechanisms (10–13). Type I interferons (IFN-α and IFN-β) bind to interferon-α/β receptor (IFNAR) on cell surfaces to induce JAK-STAT signaling and phosphorylation of STAT1 and STAT2 in an autocrine and paracrine manner. Complexes of phosphorylated STAT1, STAT2, and IRF9 enter the nucleus and induce the production of interferon-stimulated genes (ISGs). MX1 is an interferon-induced protein, and its promoter is used in our study as a luciferase reporter system to quantify the amount of IFN (14–16).

The use of RLR-binding molecules has been proposed for antiviral prophylaxis and treatment, as cancer therapy, and as vaccine adjuvants (17, 18). Hairpin RNA molecules between 67 nucleotides long (19) and 99 nucleotides long (20) were shown to have broad antiviral activity against influenza virus, dengue virus (DENV), and chikungunya virus (CHIKV) when tested in human cell lines and in mice (2). Short 5′-triphosphorylated hairpins (10 to 14 bp) have recently been demonstrated to be able to activate RIG-I mediated production of type I interferon in mice (21, 22). The antiviral activity was mediated by the induction of antiviral programs in the cells, including the production of IFN-α and -β.

DENV is an arbovirus that is transmitted to humans through the bite of an infected *Aedes* mosquito. DENV is part of the *Flaviridae* family and is a member of the *Flavivirus* genus. This family of viruses includes other viruses that are known to pose health threats to the human population globally, including yellow fever virus (YFV), West Nile virus (WNV), and Japanese encephalitis virus (JEV). DENV is an enveloped virus that contains a single-stranded, positive-sense RNA genome. This viral genome encodes a large polyprotein, which is processed by viral and host proteases into three structural proteins (capsid, prM, and envelope protein) and seven nonstructural proteins (NS1, NS2A, NS2B, NS3, NS4A, NS4B, and NS5).

The transmission of DENV involves the transfer of virus from the saliva of the biting mosquito to the dermal layer of human skin (23). The outermost, epidermal layer contains keratinocytes and Langerhans cells (LCs), which are skin-resident antigen-presenting cells (APCs) that are involved in detecting pathogens that penetrate the skin barrier (24). The dermal layer, which is located below the epidermal layer, consists of fibroblasts and immune cells, including macrophages, T cells, and dendritic cells (DCs), and is innervated with blood and lymphatic vessels that enable immune cell migration to draining lymph nodes (25). APCs are primary host cells for DENV infection (23, 26–29). Professional APCs in the skin are particularly important in the establishment of infection due to their location at the point of virus entry into the host (23, 27, 29). We have established a human skin cell assay as a model to study DC subset infection and activation *in vitro* (23). These primary skin cells are different from the conventionally used monocyte-derived dendritic cells, which are more representative of an inflammatory type of APCs and are not relevant as initial hosts. Instead, monocyte-derived dendritic cells are secondary infection targets once the infection is established (23, 29). Upon DENV infection, APCs are activated by the viral RNA binding to RIG-I and MDA5 in the cytoplasm of these cells (3).

Based on the initial work to determine the minimal RNA ligand required for interferon activation (21), we made various modifications to the original sequence and tested the ability of these newly designed immune-modulating RNAs (immRNAs) to activate the RIG-I-mediated innate immune response in host cells. We found a lead candidate immRNA, 3p10LG9, that has greater potency in activating type I interferon response than the parental construct, and we studied the protective effects of this immRNA against DENV infection both in human cell lines and in a human skin cell assay model to assess its potential as a prophylactic and therapeutic molecule.

## RESULTS

### Transfection of immRNA in human cell lines inhibits DENV-2 infection.

**FIG 1:**
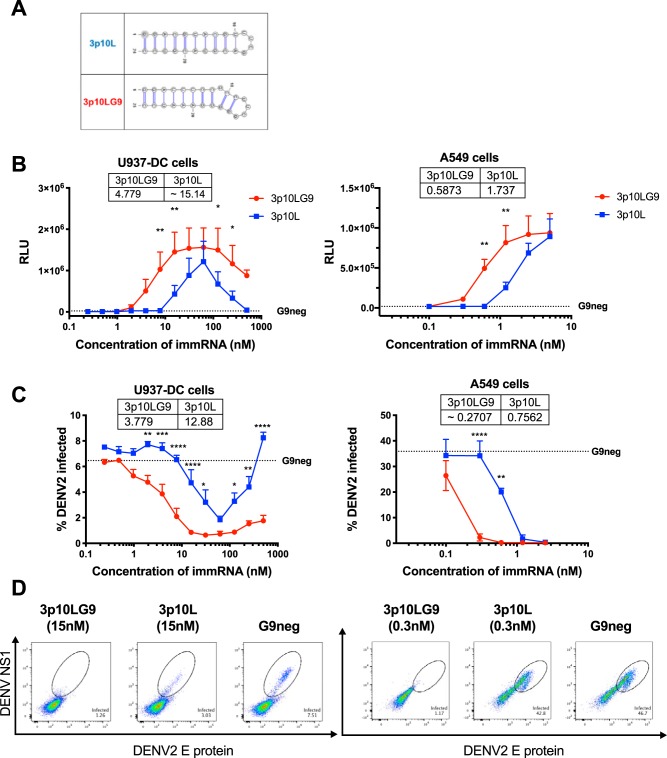
3p10LG9 induces an interferon response and is effective as prophylaxis against DENV-2 infection in both U937-DC-SIGN cells and A549 cells. (A) Structure and sequence of 3p10L and 3p10LG9. (B) 3p10L, 3p10LG9, or G9neg was transfected into U937-DC-SIGN or A549 cells. Supernatants were harvested and incubated on ISRE-luc HEK-293T reporter cells. Luminescence was measured 6 h after incubation with the supernatant. Tables show EC_50_ values (nM) for 3p10L and 3p10LG9. (C) Transfected cells were infected with DENV-2 (TSV01) at an MOI of 1 and stained with antibodies binding NS1 and the E protein (4G2) at 24 h after infection. Tables show EC_50_ values (nM) for 3p10L and 3p10LG9. (D) Representative flow cytometry graphs for infected U937-DC-SIGN cells (left) or A549 cells (right). For U937-DC experiments, values are means ± standard errors of the means (SEM) (*n* = 6) from two independent experiments. For A549 experiments, values are means ± SEM (*n* = 4) from two independent experiments. The dotted lines show the mean for the G9neg control. Statistical significance for the luciferase assay and infection assay in panels B and C was calculated for U937-DC-SIGN cells (*, *P* ≤ 0.05; **, *P* ≤ 0.01; ***, *P* ≤ 0.001; ****, *P* ≤ 0.0001) and A549 cells (**, *P* ≤ 0.01; ****, *P* ≤ 0.0001), using ordinary two-way analysis of variance (ANOVA) with Sidak’s multiple-comparison test.

The minimal length of the RIG-I-activating hairpin RNA is a 10-bp stem of a hairpin RNA, as shown previously (21). Based on that work, various modifications were made in the stem region, and the new molecules were tested for enhanced type I interferon (IFN) production in human cells compared to the original 10-bp stem construct (3p10L). One of the modified immRNA constructs, 3p10LG9, has an additional guanine nucleotide inserted at position 9 of the parental RNA construct, which forms a kink near the hairpin loop (Fig. 1A). 3p10LG9 had a significantly higher efficacy in inducing IFN production than the parental construct, 3p10L. This was seen after transfection into the monocytic cell line U937 that stably expresses DC-SIGN (U937-DC-SIGN) and into the human lung fibroblast cell line A594 (Fig. 1B). As a negative control, the 3p10LG9 construct without 5′ phosphorylation (G9neg) was used, as the 5′ phosphate group is essential for RIG-I-mediated activation of type I IFN signaling (4). In both human cell lines, 3p10LG9 activated the IFN response more efficiently than 3p10L in a dose-dependent manner (Fig. 1).

To determine if immRNA was able to inhibit DENV infection, we transfected U937-DC-SIGN cells and A549 cells with 3p10L and 3p10LG9 and infected the cells with DENV-2 at 24 h posttransfection. The percentage of infected cells was quantified by flow cytometry using E protein- and NS1 protein-specific fluorescently labeled antibodies to detect intracellular viral proteins. In both U937-DC-SIGN and A549 cells, 3p10LG9 and 3p10L reduced DENV infection in a dose-dependent manner, with 3p10LG9 being more potent than 3p10L (Fig. 1C and D). Interestingly, transfection of U937-DC-SIGN cells with either immRNA at more than 62 nM resulted in a reduced efficacy of type I interferon production and diminished antiviral effects. Overall, these results showed that 3p10LG9 had greater potency than 3p10L in inducing IFN signaling and antiviral responses against DENV2 infection in the U937-DC and A549 human cell lines.

### immRNA-mediated viral inhibition is RIG-I and type I IFN dependent.

**FIG 2:**
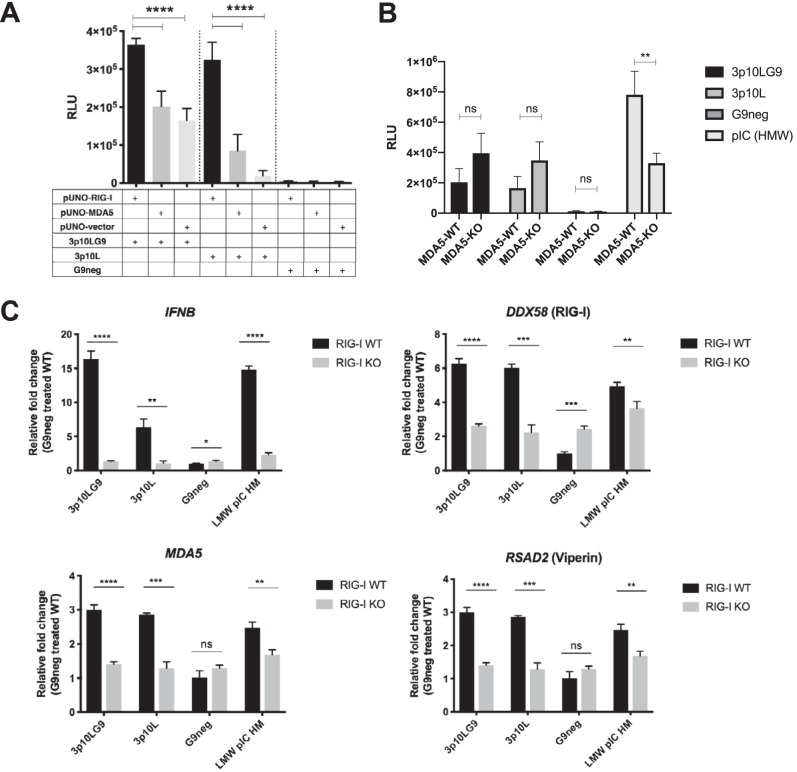
The interferon signal induced by 3p10LG9 is RIG-I dependent. (A) HEK-293T cells were transfected with 50 ng of pUNO-hRIG-I or pUNO-hMDA5. These cells were then transfected with a 10 nM concentration of either 3p10LG9, 3p10L, or G9neg. Supernatant from these transfected HEK-293T cells was incubated on HEK-293T cells containing a luciferase reporter driven by interferon-stimulated response element (ISRE-luc). Luminescence was measured at 6 h after incubation with the supernatant. Bars shown means ± standard deviations (SD). Statistical significance was determined using a one-way ANOVA test with multiple comparison (****, *P* ≤ 0.0001). (B) A549 cells (MDA5-WT) and A549-Dual KO-MDA5 cells (MDA5-KO) were transfected with 3p10LG9 (2 nM), 3p10L (2 nM), or HMW poly(I·C) (1 μg/ml), and luciferase activity was measured 24 h later by adding the supernatant to HEK293T ISRE-luc reporter cells. Data were generated by triplicate transfections from two independent experiments. Bars show means ± SEM. Statistical significance was determined with ordinary two-way ANOVA with Sidak’s multiple-comparison test (**, *P* ≤ 0.005; ns, not significant). (C) 3p10LG9, 3p10L, G9neg, or LMW poly(I·C) was transfected into either RIG-I knockout U937-DC cells (KO) or the parental U937-DC cells (WT). Gene expression analysis on mRNA extracted from transfected U937-DC cells for IFNB, DDX58 (RIG-I), MDA5, and RSAD2 (Viperin) was performed. Data are represented as fold change compared to the mean for the G9neg-treated RIG-I WT sample. Bars show means ± SD for triplicate transfections, and data are representative of two independent experiments. Statistical significance was determined using a two-tailed Student *t* test (*, *P* ≤ 0.05; **, *P* ≤ 0.01; ***, *P* ≤ 0.001; ****, *P* ≤ 0.0001; ns, not significant).

**FIG 3:**
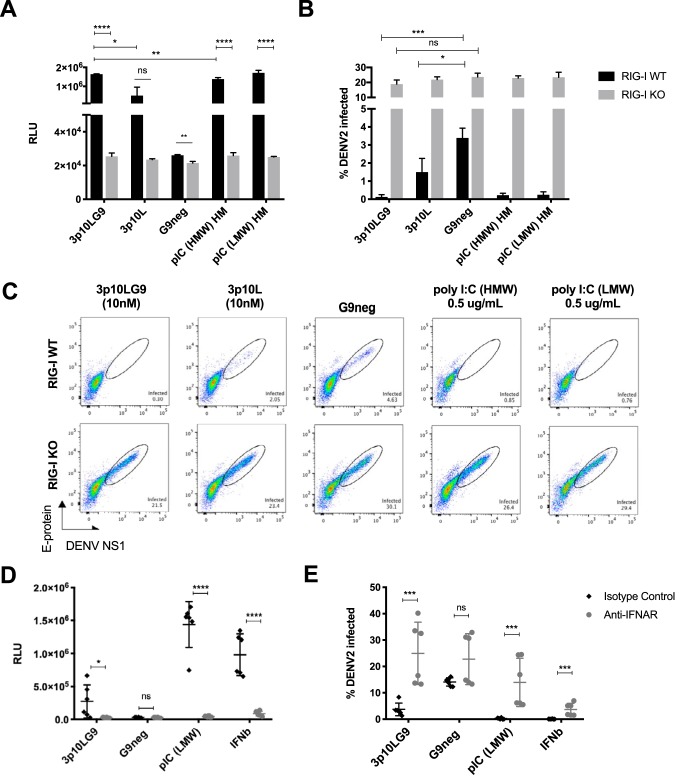
Antiviral effects of 3p10LG9 are RIG-I and IFNAR signal dependent. (A) Supernatant from U937-DC cells transfected with either immRNA or poly(I·C) was incubated on the HEK-293T cells that contain a luciferase reporter driven by an interferon-stimulated response element (ISRE-luc). Luminescence was measured at 6 h after incubation with the supernatant. Bars shown means ± SD for triplicate transfections, and data are representative of two independent experiments. Statistical significance was determined using a two-tailed Student *t* test (*, *P* ≤ 0.05; **, *P* ≤ 0.01; ***, *P* ≤ 0.001; ****, *P* ≤ 0.0001). (B) U937-DC cells pretreated with either immRNA or poly(I·C) for 24 h were infected with DENV-2 TSV01 (MOI of 1). Infected viable cells were quantified using flow cytometry with antibodies targeting NS1 and the E-protein fusion loop (4G2) at 24 h after infection. Bars show means ± SD for triplicate transfections, and data are representative of two independent experiments. Statistical significance between the treatment methods within each cell type was determined using a two-tailed Student *t* test (*, *P* ≤ 0.05; ***, *P* ≤ 0.001). (C) Representative flow cytometry graphs of viable U937-DC cells stained with antibodies binding to NS1 and the E protein (4G2). (D) U937-DC-SIGN cells were transfected with immRNA or poly(I·C) or treated with IFN-β for 6 h before the addition of 10 μg/ml of either anti-IFNAR blocking antibody or an isotype control. Bars show means ± SD from transfections done in triplicate from two independent experiments. Statistical significance was determined using a two-tailed Student *t* test (*, *P* ≤ 0.05; ****, *P* ≤ 0.0001; ns, not significant). (E) Percentage of infection in U937-DC cells from panel D after infection with DENV-2 TSV01 (MOI of 1). Bars show means ± SD for transfections in triplicate from two independent experiments. Statistical significance was determined using a two-tailed Student *t* test (***, *P* ≤ 0.001; ns, not significant).

While it has been proposed that short hairpin immRNA molecules bind to RIG-I and are unlikely to bind to MDA5 (22), we wanted to test this experimentally. We cotransfected immRNA with either RIG-I-overexpressing plasmids or MDA5-overexpressing plasmids in HEK293T cells and found that 3p10LG9 activation of IFN signaling was significantly enhanced with RIG-I overexpression. This enhancement was greater than that with MDA5 overexpression (Fig. 2A). To directly test the role of MDA5 in immRNA-mediated IFN signaling, we transfected commercially available MDA5 knockout cells (A549-Dual KO-MDA5) with immRNA. We observed that the knockout of MDA5 did not attenuate IFN production by 3p10LG9 and 3p10L (Fig. 2B). The reason why immRNA-transfected KO-MDA5 cells showed a higher IFN signal could be that A549-Dual cells secrete luciferase under the control of the interferon-stimulated gene (ISG) promoter, whereas the wild-type (WT) control cells were not modified. As a readout, IFN in the supernatant was assessed for both cell lines, as for Fig. 2A. As expected, IFN production was significantly attenuated in KO-MDA5 cells transfected with high-molecular weight (HMW) poly(I·C) compared to that in the WT-MDA5 cells. To prove that immRNA-mediated IFN activation was RIG-I dependent, we generated RIG-I knockout (RIG-I KO) U937-DC-SIGN cells using clustered regularly interspaced short palindromic repeat (CRISPR)-Cas9-mediated gene knockdown with a guide RNA (gRNA) designed to target exon 1 of human RIG-I (data not shown) and transfected these cells with immRNAs and G9neg. Interferon-stimulated gene (ISG) expression in RIG-I KO U937-DC-SIGN cells was inhibited significantly after transfection with 3p10LG9 or 3p10L (Fig. 2C). This inhibition was observed despite a slightly increased *IFNB* transcript level in G9neg (control)-treated RIG-I KO cells compared to WT cells (1.3-fold increase). *DDX58* transcript levels were still detectable despite the absence of the protein, as the primers used in the quantitative reverse transcription-PCR (RT-qPCR) were designed to a region away from exon 1, which was the target region for disruption by the gRNA. *DDX58* transcript levels were significantly higher in RIG-I KO G9neg control-treated cells than in WT cells (2.4-fold). However, this increase in the baseline levels of ISGs in the RIG-I KO cells has no significant effects on type I interferon activation, as there was no increase in the luciferase signal detected in the interferon-stimulated response element (ISRE)-luciferase assay (Fig. 3A). These results showed that 3p10LG9 was a more potent inducer of IFN-stimulated genes than the parental construct 3p10L and that upregulation of IFN and ISGs was RIG-I dependent. To determine if the antiviral effects were RIG-I dependent, we prophylactically treated WT and RIG-I KO U937-DC cells with either immRNA or poly(I·C) (low molecular weight or high molecular weight) and infected the cells 24 h later with DENV-2. Type I interferon activity was observed only with WT and not with RIG-I knockout U937-DC cells (Fig. 3A). RIG-I KO U937-DC cells showed a significantly higher percentage of DENV-2 infection than WT U937-DC cells. When pretreated with immRNA or poly(I·C), DENV replication was significantly inhibited in the WT but not in the RIG-I KO U937-DC-SIGN cells (Fig. 3B and C), suggesting that the antiviral effects observed were RIG-I dependent.

To determine if the antiviral effects observed with 3p10LG9 were type I interferon dependent, we transfected U937-DC-SIGN cells with 3p10LG9 and used an interferon alpha receptor (IFNAR)-blocking antibody to prevent IFN activation through type I interferon produced in response to RIG-I signaling. In the presence of anti-IFNAR antibody, the ISRE-induced luciferase signal was efficiently inhibited, demonstrating the functionality of the assay (Fig. 3D). Importantly, anti-IFNAR blocking antibody abolished the antiviral effects of 3p10LG9, and DENV-2 replicated as efficiently as in G9neg-treated U937-DC-SIGN cells (Fig. 3E). There was a 6-h window between transfections or addition of IFN-β and the addition of the blocking antibody. This seems to have been sufficient to trigger an antiviral response in the IFN-β-treated cells and could explain the partial rescue of DENV infection in IFN-β-treated cells by blocking antibody, compared to the more complete rescue in immRNA-treated cells. In summary, the experiments showed that the antiviral effects observed in U937-DC-SIGN cells treated with 3p10LG9 were RIG-I and type I interferon signal dependent.

### APCs from human skin transfected *ex vivo* show differential immRNA uptake and innate immune activation.

**FIG 4:**
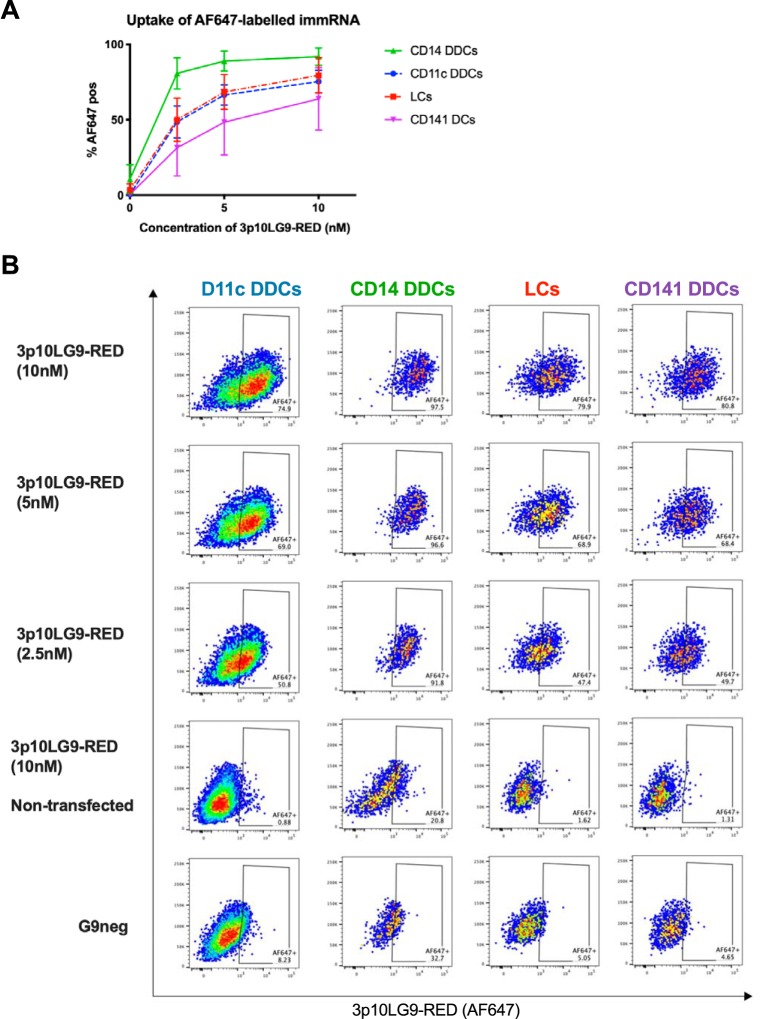
3p10LG9 uptake by primary human skin cells. (A) Skin cells were transfected with various concentrations of 3p10LG9 labeled with Alexa-Fluor 647. Cells were analyzed using flow cytometry at 24 h posttransfection. Statistical significance between the different cell types with various concentrations of 3p10LG9-RED was calculated using repeated-measures two-way ANOVA (*P = *0.0026). Values are means ± SD (*n* = 3). (B) Representative flow cytometry graphs for the data shown in panel A.

Efficient DENV infection of DCs in the skin suggests their important role in the systemic spread of DENV. Infected DCs could carry the virus from the site of infection to secondary lymphoid organs such as lymph nodes (23, 29). To test whether immRNA could block infection of primary human skin cells, healthy skin samples were processed to prepare single-cell suspensions for transfection with immRNA and subsequent flow cytometry analysis (23). We first tested which cells were most efficiently transfected with immRNA using a fluorescently labeled version of 3p10LG9 (3p10LG9-RED) that can be traced by flow cytometry. All cell types were transfectable, and uptake was most efficient in CD14^+^ dermal DCs (DDCs), followed by CD11c^+^ DDCs and Langerhans cells, with CD141^+^ DDCs having the least efficient uptake. When 3p10LG9-RED was added to the cells without transfection reagent, the uptake was minimal, demonstrating that immRNA uptake by phagocytosis was minimal (Fig. 4A and B).

**FIG 5:**
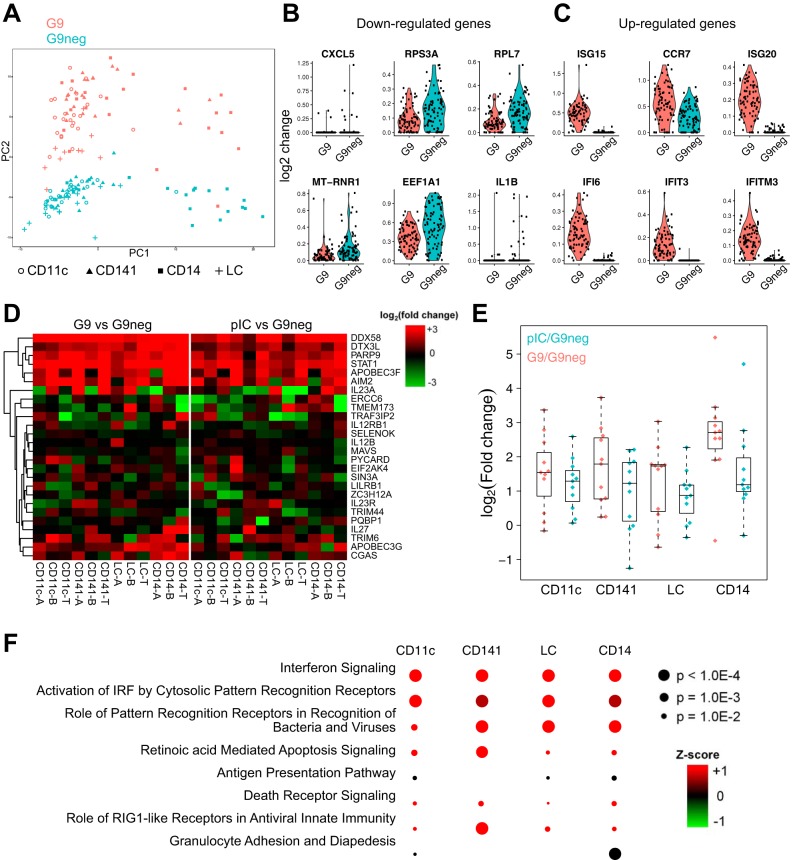
immRNA induces innate immune genes in human antigen-presenting cells. (A to C) Single-cell RNAseq analysis of combined skin APCs from one donor (see Materials and Methods for details). (A) PCA analysis of DEGs in skin cells treated with 3p10LG9 (G9) versus the same batch of cells treated with G9neg. (B) Top six downregulated genes in G9- versus G9neg-treated cells. (C) Top six upregulated genes in G9- versus G9neg-treated cells. Each symbol represents one single cell. (D to F) Bulk RNAseq analysis of combined skin APCs from five donors (see Materials and Methods for details). Suffixes -T, -B, and -A refer to individual donors. (D) Heat map display of fold changes upon stimulation of genes labeled by the gene ontology term GO:0002230, “positive regulation of defense response to virus by host.” (E) Comparison of fold changes between G9 and pIC stimulations with only the upregulated genes. (F) Ingenuity pathway analysis (IPA) of genes differentially expressed by G9 stimulation, showing the most significant overlaps between the top 10 pathways per cell type.

The activation profile of immRNA-treated was assessed by mRNA sequencing (RNAseq) of single (Fig. 5A to C) and bulk-sorted (Fig. 5D to F) skin APC subsets at 35 h after transfection. Principal-component analysis of differentially expressed genes (DEGs) in a total of 159 single APCs from one donor (combined CD11c^+^ DDCs, CD141^+^ DDCs, CD14^+^ cells, and LCs) clearly separated 3G10LG9 and G9neg-treated cells (Fig. 5A). The top six downregulated genes in 3G10LG9-treated cells included those for the chemokine CXCL5, the cytokine interleukin-1β (IL-1β), and ribosomal proteins (Fig. 5B). The top six upregulated genes comprised five interferon-induced genes and the gene for immune cell-homing chemokine receptor CCR7 (Fig. 5C). To further assess transcriptome changes after immRNA activation in more than one donor, bulk-sorted skin APC subsets from five donors were sequenced. Similar to the findings from the single-cell analysis (Fig. 5A), CD14^+^ cells were transcriptionally distinct from the other APC subsets (data not shown). Despite this, there was a high overlap of DEGs between cell types, showing that at least part of the immRNA-mediated activation was common to all skin APC subsets. At the same time, several of the top 12 DEGs identified in the single-cell analysis were confirmed in the bulk-cell analysis (data not shown). Heat maps of DEGs selected based on a defined set of genes associated with antiviral responses in host cells showed that various genes were upregulated for both 3p10LG9- and pIC-treated cells (Fig. 5D). 3p10LG9 appeared to be a generally stronger activator of antiviral host response genes than pIC (Fig. 5E). This might be related to cell-type-specific expression levels of RIG-I and Toll-like receptor 3 (TLR3), the ligands of immRNA and pIC, respectively. Ingenuity pathway analysis (IPA) of DEGs per cell type revealed that the top three pathways were common for the individual APC subsets. However, other pathways were more cell type specific, such as the “role of RIG-I like receptors in antiviral innate immunity,” which was more significant in CD141^+^ cells. In turn, this cell type was not associated with the antigen presentation pathway (Fig. 5F).

These data emphasized that immRNA efficiently activates antiviral transcriptional programs in primary human APCs. This is important because APCs are known for their key roles in antiviral responses during natural infection.

### Prophylactic and therapeutic antiviral efficacy of immRNA in *ex vivo* human APCs.

**FIG 6:**
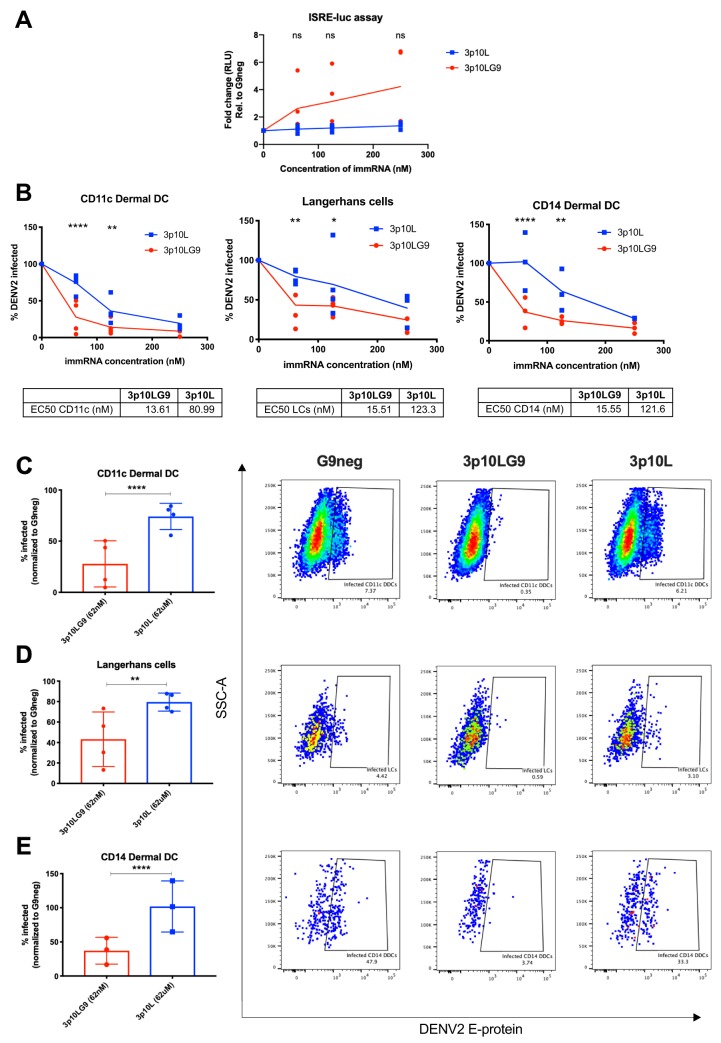
3p10LG9 has a higher efficacy than 3p10L as a prophylaxis against DENV-2 infection in primary human skin DCs. (A) Human skin DCs were transfected with 250 nM, 125 nM, and 62 nM 3p10LG9 or 3p10L and incubated for 24 h. Supernatant was incubated on HEK-293T cells containing a luciferase reporter driven by an interferon-stimulated response element (ISRE-luc), and luminescence was measured after 6 h. Results are presented as fold change compared to G9neg. Each symbol represents a sample from one donor. Statistical significance (*P < *0.05) was determined using a two-way ANOVA with Dunnett’s multiple-comparison test (ns, not significant). (B) Human skin DCs transfected with 250 nM, 125 nM, and 62 nM 3p10LG9 or 3p10L for 24 h were infected with DENV-2 at an MOI of 5 for 48 h. Flow cytometry was used to quantify the percentage of DENV-2-infected cells in each subpopulation of skin DCs by intracellular staining with 4G2 antibody. The percentage of cells infected for each condition was normalized to G9neg. (C to E) Percentage of infected cells for each subpopulation of skin DCs taken from the data plotted in panel B after transfection with 62 nM 3p10LG9 or 3p10L (left), and representative flow cytometry (right). (C) CD11c DDCs; (D) Langerhans cells; (E) CD14 DDCs. Each symbol represents one donor. Bars show means ± SD. Statistical significance (*P < *0.05) was determined using a two-way ANOVA with Dunnett’s multiple-comparison test (*, *P* ≤ 0.05; **, *P* ≤ 0.01; ****, *P* ≤ 0.0001).

Next, we tested whether human skin APCs treated with immRNA were protected from DENV infection. To do this, we treated skin single-cell suspensions with immRNA at various concentrations. Twenty-four hours later, we collected the supernatants for ISRE-luciferase assay and infected the cells with DENV at a multiplicity of infection (MOI) of 5. At 48 h after infection, the percentage of infected, DENV E protein-containing skin APCs was quantified by flow cytometry (23). Human skin APCs that received prophylactic treatment with 3p10LG9 induced type I IFN more efficiently than those treated with 3p10L, based on the ISRE-luciferase assay (Fig. 6A). Prophylactic treatment of human skin APCs with immRNA also protected the cells from DENV infection in a dose-dependent manner. Fifty percent effective concentration (EC_50_) values showed that 3p10LG9 was more potent in inducing an antiviral response than 3p10L in CD11c DDCs (3p10LG9, 13.6 nM; 3p10L, 81.0 nM), LCs (3p10LG9, 15.5 nM; 3p10L, 123.3 nM), and CD14 DDCs (3p10LG9, 15.5 nM; 3p10L, 121.6 nM) (Fig. 6B). At the lowest concentration tested (62 nM), 3p10LG9 was significantly more effective than 3p10L in reducing the number of infected CD11c DDCs (*P ≤ *0.01) and CD14 DDCs (*P ≤ *0.05) (Fig. 6C to E).

**FIG 7:**
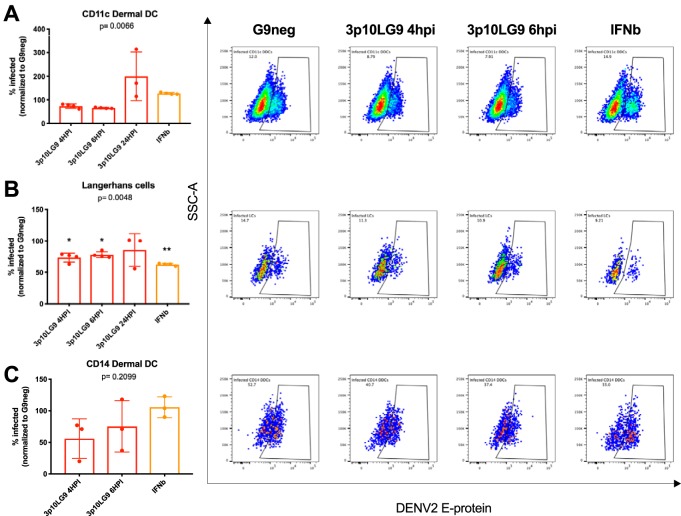
3p10LG9 has a moderate therapeutic effect against DENV-2 infection in primary human Langerhans cells. DENV-2-infected human skin DCs were transfected with 62 nM 3p10LG9, 3p10L, or G9neg at the indicated time points postinfection. Graphs showing the percentage of infected cells within each subpopulation of skin DCs (left) reflect staining with 4G2 antibody, as represented by the flow cytometry graphs on the right. The percentage of cells infected under each condition was normalized to the G9neg control per donor. (A) CD11c dermal DCs; (B) Langerhans cells; (C) CD14 dermal DCs. Each dot represents one donor. Statistical significance was determined using a one-way ANOVA with Dunnett’s multiple-comparison test (*, *P ≤ *0.05; **, *P ≤ *0.01; ***, *P ≤ *0.005).

To determine if immRNA can act as a therapeutic for DENV-infected skin APCs, we infected skin single-cell suspensions with DENV at an MOI of 5 and treated these cells with 62 nM 3p10LG9 at 4, 6, and 24 h after infection. Cells were stained at 48 h after infection for flow cytometry-based quantification of infection. Since the infection efficacy varied up to 40% between individual skin samples, the infection was normalized to the G9neg control from the 4-h-postinfection time point. The inhibitory effect of 3p10LG9 overall was modest. A significant reduction of infected cells after treatment with 3p10LG9 was seen only in Langerhans cells and only at early time points of 4 h and 6 h postinfection (Fig. 7B). Small therapeutic effects were seen in the CD11c DDCs (Fig. 7A) and CD14 DDCs (Fig. 7C) at the early time points. After treatment with 3p10LG9 at 24 h after infection, the percentage of infected CD11c^+^ DDCs cells 48 h later tended to be higher than that for the G9neg-treated cells. It is possible that the virus inhibited the antiviral response more efficiently in this cell subset, subverting the activity of RIG-I ligand. Overall, these data suggest that 3p10LG9 had a modest therapeutic antiviral effect on DENV-2 infection in primary human skin APCs.

## DISCUSSION

RIG-I-like receptors (RLRs) have been known to play an important role in sensing viral infection and activating antiviral immune response, including the production of type I interferon and proinflammatory cytokines (1, 3).

Through an initial screen we found that a kink in the 9th nucleotide from the 5′ end generated by the addition of a guanine nucleotide enhanced type I interferon activation 6-fold (Fig. 1). It has been shown by others that various structural modifications to 5′-pppRNA were able to enhance RIG-I-mediated activation of type I interferon and antiviral activity. In particular, longer sequences as well as the addition of poly(U) sequences along the stem of the RNA duplex has been shown to enhance immune activation (20, 21).

An assessment of differential binding of 3p10L and 3p10LG9 to RIG-I using hydrogen-deuterium exchange coupled to mass spectrometry (HDX-MS) was carried out and will be published separately. The findings suggest that a stronger RNA-protein interaction leads to more exposed caspase activation and recruitment domains (CARDs) and thus increased RIG-I mediated signaling with 3p10LG9 (H. Y. Yong et al., submitted for publication). Our findings, together with the findings from others, suggest that the structural and sequential features of the RNA species play an important role in activating type I interferon responses in host cells. This knowledge can be used to further improve the drug-like properties of the RIG-I ligands.

Proof of activity of immRNA in human cells is crucial for a potential therapeutic application. We had previously established a human skin cell assay as a model to study the infection of various DC subsets *in vitro* (23). We reported that the main DC subsets susceptible to DENV infection were the CD11c^+^ dermal DCs, CD14^+^ dermal DCs, and Langerhans cells. While all skin DCs were receptive to transfection with immRNA, uptake by CD14^+^ cell was most efficient. The CD14^+^ DDC population is transcriptionally and functionally related to human monocytes and macrophages, which have a higher phagocytic capacity than conventional DCs (30). Phagocytosis activity can possibly improve the transfection efficacy. We showed that human skin cells pretreated with immRNA were effectively primed through type I interferon production and upregulation of interferon-stimulated genes (ISGs). This resulted in the inhibition of DENV replication for the three virus-susceptible DC subsets. Using U937-DC-SIGN cells, we demonstrated that the immRNA-mediated antiviral effect was both RIG-I and type I interferon dependent (Fig. 2 and 3). We also showed that the lack of MDA5 did not significantly attenuate the IFN activation by immRNA, suggesting that type I IFN signaling induction was MDA5 independent. These findings are in line with previous results showing that minimal-length dsRNA molecules bind to RIG-I to initiate type I interferon signaling (21, 31, 32).

Therapeutic treatment appeared to only moderately decrease DENV replication in Langerhans cells but not in the other skin APC subsets. LCs are located in the epidermis, the most superficial layer of the skin. It is still unclear to what extent Langerhans cells come into contact with the virus once the host is bitten by an infected mosquito, since the probing for blood vessels is in the dermis (23, 27, 33). Regardless, our results suggest that Langerhans cells are possibly more sensitive to RIG-I-mediated innate immune activation than CD11c^+^ DDCs or CD14^+^ DDCs after DENV infection has been established. More work is required to determine the factors involved in the responsiveness of Langerhans cells to RIG-I-mediated immune activation. One limitation of the assay is the relatively high MOI (5) required to achieve detectable levels of DENV E protein by flow cytometry. More-sensitive approaches for virus detection, such as viral RNA sequencing on sorted DC subsets, could give us better insight into the therapeutic effects of immRNA and RIG-I-mediated immune activation, as well as determining the factors involved in the responsiveness of Langerhans cells to RIG-I-mediated immune activation.

The concentration-dependent activity of 3p10LG9 observed in U937-DC-SIGN cells could indicate negative feedback inhibition of interferon signaling as a result of overstimulation at high immRNA concentrations. Using a mouse model with transgenic expression of picornaviral RNA-dependent RNA polymerase (RdRP), Painter et al. (34) found that the interferon-stimulated genes (ISGs) were up to 300-fold elevated in mouse tissues and that this elevated ISG profile protected RdRP mice from viral infection. Interestingly, these RdRP mice were healthy with normal longevity despite life-long, constitutive MDA5-mediated innate immune system activation caused by the presence of endogenous long dsRNA. Genes involved in the negative regulation of type I IFN signaling, such as USP18, NLRC5 and LGP2, were upregulated in the gene expression data (34). In addition to the results with U937-DC-SIGN cells in our study, a potential negative regulation of the antiviral effects after the establishment of a viral infection was observed for primary cells. CD11c^+^ DCs showed a trend for higher infection when treated with immRNA only 24 h after infection (Fig. 7). This time- and concentration-dependent negative feedback loop could be important to limit inflammation and cell death. Accordingly, no cell death was observed in the U937-DC-SIGN cells, even at the highest immRNA concentration. Similarly, primary skin APCs were unaffected by high concentrations of immRNA. Death receptor signaling-related DEGs were not among the top hits in the RNAseq analysis (Fig. 5F). This was different for the A549 fibroblast cell line, which did not survive transfection with immRNA at high concentrations. The induction of apoptosis and cell death is similar to what others have described when using a RIG-I agonist in A549 cells (2, 19). The wider active window of 3p10LG9 compared to 3p10L shown in U937 cells (Fig. 1) could be a key advantage for a potential therapeutic application.

It was surprising that 3p10LG9 appeared to activate skin APCs more efficiently than poly(I·C) (Fig. 5E), given that poly(I·C) can bind to multiple receptors (RIG-I, MDA5, and TLR3), whereas 3p10LG9 binds only to RIG-I. However, it is difficult to directly compare the efficacy of short RNA molecules like 3p10LG9 with that of poly(I·C), given the large difference in their molecular weight and potential differences in transfection efficiency. Nevertheless, it is worth noting that 10 nM 3p10LG9 is equivalent to a concentration of about 0.08 μg/ml, much less than the concentration of 0.5 μg/ml poly(I·C) used in the cell line experiments (Fig. 2 and 3). Given the key role of RIG-I signaling activation at the interface of innate and adaptive immune responses (35, 36), RIG-I signaling in tissue-resident APCs as a physiologically relevant model should be further studied.

In summary, we have shown that the minimal RNA ligands are capable of generating an effective innate immune response in host cells with natural infection, and this response inhibits DENV replication in primary cells efficiently when used as a prophylaxis. Beyond dengue infection, our findings could be relevant for topical or systemic application of RNA-based ligands targeting RIG-I and for the ensuing responses in general.

## MATERIALS AND METHODS

### *In vitro* synthesis of immRNA.

RNAs were transcribed with annealed primer pairs containing the T7 promoter with chemically synthesized DNA from IDT. Reaction were carried out in a mixture of 40 mM Tris HCl buffer (pH 7.9), 30 mM MgCl_2_, 2 mM spermidine, 10 mM dithiothreitol (DTT), 0.01% Triton X-100, 5 to 6 mM GTP, 4 mM nucleoside triphosphates (CTP, ATP, and UTP), 1 μM annealed DNA template, 400 nM T7 RNA polymerase, and 0.2 U/ml thermostable inorganic pyrophosphatase for 16 h at 37°C. Transcribed RNAs were purified by phenol-chloroform-isoamyl alcohol (25:24:1, vol/vol) extraction followed by ethanol precipitation. The RNA pellet was resuspended in 10 mM HEPES buffer (pH 7.4) and subjected to further purification with a HiTrap Q HP column. The eluted RNAs were subjected to ethanol precipitation and further purified from 20% denaturing PAGE followed by ethanol precipitation. Purified RNAs were resuspended in ME 50 buffer (10 mM morpholinepropanesulfonic acid [MOPS] [pH 7], 1 mM EDTA, and 50 mM NaCl). The sequences for the RNA were GGACGUACGUUUCGACGUACGUCC for 3p10L and GGAUUUCCGCCUUCGGGGGAAAUCC for 3p10LG9.

### Ethics statement.

Healthy human skin tissue was obtained from mastectomy surgery. The study was approved by the institutional review board (National Health Group Domain Specific Review Board [NHG DSRB 2015/00725 and 2017/00812]), and patients gave written informed consent. All skin samples were processed on the day of surgery.

### Human skin DC isolation.

Protocols for isolating single cells from human skin were described in detail previously (23). For the isolation of human skin cells, 300-mm dermatome sections were incubated in RPMI plus 10% heat-inactivated fetal bovine serum (FBS) (Gibco) containing 0.8 mg/ml collagenase (type IV; Worthington Biochemical) and 0.05 mg/ml DNase I (Roche) for 12 h. After incubation, cells were filtered through a 70-μm strainer to obtain a single-cell suspension.

### Cell lines.

HEK-293T, U937, and A549 cells (ATCC) were grown in RPMI supplemented with 10% FBS (Gibco). U937 cells expressing DC-SIGN were generated by lentiviral transfection (37). A549-Dual KO-MDA5 cells were purchased from InvivoGen and grown in Dulbecco modified Eagle medium (DMEM) supplemented with 10% FBS (Gibco). This cell line was generated to express the secreted Lucia luciferase, and the reporter gene is under the control of an ISG54 minimal promoter in conjunction with five IFN-stimulated response elements. HEK-293T cells expressing MX1P-luc were generated by Georg Koch (38) (University of Freiburg, Germany) and were a kind gift from Matthias Habjan (Max Planck Institute of Biochemistry, Germany). HEK-293T cells containing the ISRE-luc reporter plasmid was generated by transfecting 0.5 μg of plasmid using the 293fectin transfection reagent (Thermo Fisher Scientific). The ISRE-luc plasmid used to transfect HEK-293T cells was a kind gift from Matthias Habjan.

RIG-I knockout cell lines were generated by lentivirus transduction of U937-DC cells with plasmid pRRL-gRNA-Cas9-T2A containing a gRNA sequence targeting exon 1 of RIG-I. The RIG-I gRNA-containing plasmid was a kind gift from Alvin Tan (Genome Institute of Singapore, A*STAR, Singapore). Lentiviral particles were produced on 293T cells by using the 293fectin transfection reagent (Thermo Fisher Scientific) with the following three plasmids: (i) pMDLg/pRRE, which includes *gag* (coding for the virion main structural proteins), *pol* (which is responsible for the retrovirus-specific enzymes), and RRE (a binding site for the Rev protein, which facilitates export of the RNA from the nucleus); (ii) pRSV-Rev, carrying the HIV-1 *rev* under the transcriptional control of a Rous sarcoma virus (RSV) U3 promoter; and (iii) pMD2.G, a vesicular stomatitis virus (VSV) G envelope-expressing plasmid. pMDLg/pRRE (Addgene number 12251), pRSV-Rev (Addgene number 12253), and pMD2.G were generated by Didier Trono (Lausanne, Switzerland). Successfully transduced cells were selected by supplementing the culture medium with 2 μg/ml puromycin. Genomic DNA was extracted from cells by using a “HotSHOT” genomic DNA preparation method described previously (39). Purified DNA was sent for sequencing (First Base) using primers which flank exon 1 (forward, 5′-GGAGGGAAACGAAACTAGCC-3′; reverse, 5′-GCTCCTCAAACTCTGGCAAC-3′). Sequences were compared with the publicly available sequence for human DDX58 on Ensembl (ENSG00000107201.9).

### Virus.

DENV-2 strain TSV01 (NCBI accession number AY037116.1), used for infection experiments in human cell lines, is a patient isolate that has been passaged in C6/36 mosquito cells for 5 to 20 passages. D2Y98P, used in the infection of primary human skin DCs, was derived from an infectious clone. The enhanced viral RNA synthesis of D2Y98P was mapped to a natural mutation in the NS4b protein, and this mutation had no effect on the IFN-inhibiting capacity of the virus (40).

### RNA screening with type I IFN bioassay.

HEK-293T MX1P-luc cells were seeded into white 96-well plates at a density of 2.5 × 10^4^ cells per well and incubated overnight. immRNA was diluted to the appropriate concentrations and transfected with 293fectin transfection reagent (Thermo Fisher Scientific) according to the manufacturer’s instructions. Cells were incubated for 24 h and then lysed and analyzed using the Bright-Glo luciferase assay system (Promega) on a GloMax-Multi microplate reader (Promega) according to the manufacturer’s instructions.

### qPCR.

U937-DC-SIGN cells were seeded in a 24-well plate at a density of 3.0 × 10^5^ cells per well in 500 μl of RPMI with 10% FBS and incubated overnight. immRNA was diluted to the appropriate concentrations and transfected (in triplicate) with Hilymax (Dojindo Molecular Technologies) according to the manufacturer’s instructions. After 24 h of incubation, cells were centrifuged at 500 × *g* for 4 min and harvested in TRIzol reagent (Thermo Fisher Scientific), and total RNA was harvested according to the manufacturer’s instructions. RNA was reverse transcribed using the SuperScript VILO cDNA synthesis kit (Invitrogen). PCR primers were purchased from Integrated DNA Technology, and quantitative RT-PCR was performed on an ABI 7900 HT real-time PCR system (Applied Biosystems) using iTaq Universal SYBR green Supermix (Bio-Rad Laboratories). Primer sequences can be found in Table 1. Analysis of quantitative PCR (qPCR) data was done by relative quantitation by the ΔΔ*C_T_* method using the beta-actin gene as the reference gene control.

**TABLE 1:** Sequences of primers used for RT-qPCR

Gene	Species	Sense	Sequence (5′ to 3′)
*IFNB*	Human	Sense	CTCTCCTGTTGTGCTTCTCC
*IFNB*	Human	Antisense	GTCAAAGTTCATCCTGTCCTTG
*ACTB*	Human	Sense	TCGTGCGTGACATTAAGGAG
*ACTB*	Human	Antisense	GTCAGGCAGCTCGTAGCTCT
*DDX58*	Human	Sense	GCCATTACACTGTGCTTGGAGA
*DDX58*	Human	Antisense	CCAGTTGCAATATCCTCCACCA
*RSAD2*	Human	Sense	CACAAAGAAGTGTCCTGCTTGGT
*RSAD2*	Human	Antisense	AAGCGCATATATTCATCCAGAATAAG

### Bioassay for type I IFN production.

Supernatant from immRNA-transfected cells was incubated on HEK-293T cells that had been transfected the day before with 0.5μg of ISRE-luc plasmid and plated in a 96-well white opaque plate the next day. Supernatant was incubated for 6 h before being lysed and analyzed by using the Bright-Glo luciferase assay system (Promega) on a GloMax-Multi microplate reader (Promega) according to the manufacturer’s instructions.

### Type I IFN bioassay for RLR-expressing HEK-293T cells transfected with immRNA.

HEK-293T cells were seeded in a 24-well plate at a density of 1.25 × 10^5^ cells per well in RPMI with 10% FBS, and then 50 ng of pUNO-hRIG-I or pUNO-hMDA5 (31) was transfected using Lyovec (InvivoGen) and cells were incubated overnight. HEK-293T cells expressing RLRs were transfected with immRNA. Supernatant from cells was harvested 24 h after transfection with dsRNA, and a type I interferon bioassay using HEK-293T cells expressing ISRE-luc was done.

### U937-DC-SIGN cell IFNAR blocking assay.

U937-DC-SIGN cells were seeded in a 96-well plate at a density of 0.6 × 10^5^ cells per well and transfected with immRNA (in triplicate) with Hilymax (Dojindo Molecular Technologies) according to the manufacturer’s instructions. After 6 h, anti-human IFNAR blocking antibody (clone MMHAR-2; PBL Interferon Source) or an IgG isotype control (R&D Systems) was added at a concentration of 10 μg/ml. After overnight incubation, supernatant was harvested and a bioassay for type I interferon was done. U937-DC-SIGN cells were infected with DENV-2 (TSV01) at an MOI of 1, and infection was quantified.

### DENV-2 infection and flow cytometry analysis.

U937-DC-SIGN cells were seeded in a 96-well plate at a density of 0.6 × 10^5^ cells per well and transfected with immRNA (in triplicate) using Hilymax (Dojindo Molecular Technologies) according to the manufacturer’s instructions. A549 cells were seeded in a 96-well plate at a density of 1.0 × 10^4^ cells per well and transfected with immRNA using the 293fectin transfection reagent (Thermo Fisher Scientific) according to the manufacturer’s instructions. After 24 h of incubation, transfected U937-DC-SIGN and A549 cells were infected with DENV-2 (TSV01 strain) at MOIs of 1 and 5, respectively. Cells were incubated with RPMI containing DENV-2 for 2 h. After two washes, infected cells were resuspended in RPMI with 10% FBS and placed in the incubator for 24 h. For fluorescence-activated cell sorter (FACS) analysis, washed cells were fixed and permeabilized by resuspending cells in Cytofix/Cytoperm buffer (BD Biosciences). Dengue virus E protein was stained with anti-E protein antibody (4G2) (ATCC) conjugated to Alexa 647 and anti-NS1 antibody conjugated to Alexa 488. Fluorescence on these cells was measured on a BD FACS Canto II Analyzer (BD Biosciences), and analysis was done on FlowJo (Tree Star Inc.). Cells that stained positive for both NS1 and E protein were considered infected.

Human skin DCs were isolated and transfected with immRNA as described previously (23). For prophylactic studies, isolated human skin DCs were infected with DENV-2 (D2Y98P strain) at an MOI of 5 at 24 h posttransfection. For therapeutic studies, isolated human skin DCs were infected with DENV-2 (D2Y98P strain) at an MOI of 5, and transfection of immRNA was done at 4 h, 6 h, and 24 h postinfection. Infected cells were analyzed at 72 h postinfection to determine the percentage of infected cells using flow cytometry. Human recombinant IFN-β (1,000 U; Immunotools) was added to cells at 4 h postinfection. Flow cytometry was performed on an LSRII (Becton Dickinson [BD]), and data were analyzed using FlowJo (Tree Star Inc.). The following reagents for staining of human skin DCs were used: fixable live/dead dye (Thermo Fisher Scientific); anti-CD1a (HI149) (BioLegend); anti-CD11c (B-ly6), anti-CD45 (HI30), and anti-HLA-DR (L243) (all from BD Biosciences); anti-CD141 (AD5-14H12) (Miltenyi); anti-CD14 (RMO52) (Beckman Coulter); and anti-E protein (4G2) (ATCC) conjugated to Alexa 647.

### RNAseq experiments.

**(i) Single-cell RNAseq.** Skin cell subsets were identified as described in “DENV-2 infection and flow cytometry analysis” above, sorted individually into 96-well PCR plates, and frozen immediately. Single cells were processed using the SMARTseq2 protocol (41), with the modifications that 1mg/ml bovine serum albumin (BSA) lysis buffer (Ambion; Thermo Fisher Scientific, Waltham, MA, USA) and 200 pg cDNA with 1/5 reaction of the Illumina Nextera XT kit (Illumina, San Diego, CA, USA) were used.

The length distribution of the cDNA libraries was monitored using a DNA high-sensitivity reagent kit on the Perkin-Elmer LabChip (Perkin-Elmer, Waltham, MA, USA). All samples were subjected to an indexed paired-end sequencing run of 2 × 151 cycles on an Illumina HiSeq 4000 system (Illumina, San Diego, CA, USA) (309 samples/lane).

Pair-end raw reads were aligned to the human reference genome using RSEM version 1.3.0 (42). The human reference genome GRCh38 version 25 release by Gencode was used (https://www.gencodegenes.org/human/release_25.html). Transcript-per-million-read (TPM) values were calculated using RSEM version 1.3.0 (42) and were log transformed [log_2_(expression + 1)] for downstream analysis. Quality control, selection of highly variable genes, principal-component analysis (PCA), and differential gene analysis were performed using Seurat R package version 2.0 (43). Low-quality cells from our data set were filtered out based on a threshold for the number of genes detected (a minimum of 200 unique genes per cell), and all genes that were not detected in at least 1.9% of all of our single cells were discarded, leaving 159 cells and 15,174 genes for all further analyses. PCA was performed on the 810 highly variable genes after scaling the data. Differential gene expression was analyzed using the negative bimodal Wald test, selecting genes with an adjusted *P* value (with Benjamini-Hochberg correction) for the estimated fold changes of <0.05.

**(ii) Bulk RNAseq.** Five hundred cells were sorted per subset and donor, and RNA was isolated using PicoPure RNA isolation kits. cDNA libraries were prepared as described previously (41) with the following modifications: (i) 1 mg/ml BSA lysis buffer (Ambion; Thermo Fisher Scientific, Waltham, MA, USA), (ii) addition of 20 μM template-switching oligonucleotide (TSO), and (iii) use of 200 pg cDNA with 1/5 reaction of the Illumina Nextera XT kit (Illumina, San Diego, CA, USA).

The length distribution of the cDNA libraries was monitored using a DNA high-sensitivity reagent kit on the Perkin-Elmer LabChip (Perkin-Elmer, Waltham, MA, USA). All samples were subjected to an indexed paired-end sequencing run of 2 × 151 cycles on an Illumina HiSeq 4000 system (Illumina, San Diego, CA, USA) (32 samples/lane).

Paired-end reads with a length of 150 bp (300 bp for a pair) were mapped to the human transcriptome sequences obtained from Gencode version 29 (44) using Salmon (version 0.11.3) (45). Transcript-wise read counts obtained from Salmon were summarized to gene-wise counts using the tx2gene R/Bioconductor package (46). Gene-wise summarized counts for samples relevant to the conditions being compared were loaded into DESeq2 (47). Genes with at least one count in at least one sample were retained in the data set. Using DESeq2, the count data were fitted to a negative binomial generalized linear model. Size factors for library size normalization and the mean and dispersion parameters for each gene were estimated using the estimateSizeFactors and estimateDispersion functions. Differential gene expression was analyzed using the negative binomial Wald test. The *P* values for the estimated fold changes were corrected for multiple testing using the Benjamini-Hochberg method, and differentially expressed genes were selected based on an adjusted *P* value of <0.05. Lists of genes identified as differentially expressed upon G9 stimulation in each cell type were supplied to ingenuity pathway analysis (IPA) software along with the respective fold changes and *P* values. Pathway enrichment analysis based on differential expression was performed in IPA for determining pathways that are significantly modulated by stimulation in each cell type.
